# Computational analysis to define efficacy & molecular mechanisms of 7, 4’- Dihydroxyflavone on eosinophilic esophagitis: *Ex-vivo* validation in human esophagus biopsies

**DOI:** 10.3389/fimmu.2022.1015437

**Published:** 2022-12-15

**Authors:** Anish R. Maskey, Zhen-Zhen Wang, Xin Chen, David Dunkin, Nan Yang, Gary Soffer, Qian Yuan, Xiu-Min Li

**Affiliations:** ^1^ Department of Pathology, Microbiology & Immunology, New York Medical College, Valhalla, NY, United States; ^2^ Academy of Chinese Medical Sciences, Henan University of Chinese Medicine, Zhengzhou, China; ^3^ Department of Pediatrics, Icahn School of Medicine at Mount Sinai, New York, NY, United States; ^4^ General Nutraceutical Technology, Elmsford, NY, United States; ^5^ Department of Allergy and Immunology, Yale University, New Haven, CT, United States; ^6^ Food Allergy Center, Massachusetts General Hospital, Harvard Medical School, Boston, MA, United States; ^7^ Department of Otolaryngology, Westchester Medical Center, New York Medical College, Valhalla, NY, United States

**Keywords:** 7, 4 dihydroxy flavone (DHF), eosinophilic esophagitis, anti-inflammation, computational modelling, molecular docking

## Abstract

**Introduction:**

Eosinophilic Esophagitis (EoE) is a chronic condition characterized by eosinophilic inflammation of the esophagus which leads to esophageal dysfunction with common symptoms including vomiting, feeding difficulty, dysphagia, abdominal pain. Current main treatment options of EoE include dietary elimination and swallowed steroids. Diet elimination approach could lead to identifying the trigger food(s), but it often requires repeated upper endoscopy with general anesthesia and potentially could negatively affect nutrition intake and growth of the child and individuals’ quality of life. Although the swallowed steroid treatment of effective, the EoE will universally recur after discontinuation of the treatment. Digestive Tea formula (DTF) has been used by the Traditional Chinese Medicine (TCM) practice to improve GI symptoms in EoE patients, including abdominal pain, GE reflux, and abnormal bowel movement. Previously, a flavonoid small molecule compound 7, 4 dihydroxy flavone (DHF) from *Glycyrrhiza uralensis* in DTF inhibited eotaxin, Th2 cytokine and IgE production *in vitro* and *in vivo*.

**Method:**

This study comprehensively evaluates the potential therapeutic and immunological mechanisms underlying DHF improvement of symptoms related to EoE using computational modeling, including target mining, gene ontology enrichment, pathway analyses, protein-protein interaction analyses, in silico molecular docking and dynamic simulation followed by ex-vivo target validation by qRT-PCR using cultured human esophagus biopsy specimen with or without DHF from patients with EoE.

**Results:**

Computational analyses defined 29 common targets of DHF on EoE, among which TNF-α, IL-6, IL1β, MAPK1, MAPK3 and AKT1 were most important. Docking analysis and dynamic simulation revealed that DHF directly binds TNF-α with a free binding energy of -7.7 kcal/mol with greater stability and flexibility. Subsequently, in the human esophagus biopsy culture system, significant reduction in levels of TNF-α, IL-6, IL-8 and IL1-β was found in the supernatant of biopsy sample cultured with DHF. Furthermore, the gene expression profile showed significant reduction in levels of TNF-α, IL1-β, IL-6, CCND and MAPK1 in the esophagus biopsy sample cultured with DHF.

**Discussion:**

Taken together, the current study provides us an insight into the molecular mechanisms underlying multi-targeted benefits of DHF in the treatment of EoE and paves the way for facilitating more effective EoE therapies.

## Introduction

EoE is a chronic, immune/antigen- mediated allergic disease of the esophagus that is characterized clinically by esophageal dysfunction and histologically by eosinophil-predominant inflammation (> 15 eosinophils/HPF). The common symptoms of esophageal dysfunction include vomiting, feeding difficulty, dysphagia, and abdominal pain. The incidence of EoE is increasing with estimated prevalence in the US ranging from 40-90 cases/100000 (1). EoE predominates in Caucasian (81%) (2) and middle-aged (30–40 years) men (3) with a male-to-female ratio approaching 3:1 (4). The potential mechanism of EoE pathogenesis includes over expression of Th2 cytokines, genetic predisposition, and environmental stimulation, which initiate esophageal inflammation and subsequent tissue remodeling and fibrosis. It is possible that other molecular targets exist and yet to be identified and studied to better understand the complexity of EoE pathogenesis.

The current therapy for EoE includes the use of swallowed steroid and dietary elimination (5). The dietary approach is proven to be effective in 90% EoE patients and can lead to identifying the diet trigger(s). Sustained remission can be achieved by permanently eliminating the triggers. But multiple upper endoscopies under general anesthesia are often required and prolonged diet avoidance could negatively affect the child’s nutrition intake, physical growth, and quality of life. Systemic steroids although show significant effect in reducing esophageal eosinophils, long-term use is associated with serious side effect. Swallowed steroid treatment is effective and of less side effect, but relapse of EoE is universal after the treatment is stopped (6). Proton-pump inhibitors (PPI) are effective in 15-30% of EoE patients who are PPI-responsive, but long-term use of PPI is associated with a number of side effect (7). Several humanized antibody therapies have been designed to block IL-5 and IL-13 and have showed promising effect in treating EoE (8). These specific therapies are likely to target only particular aspects of the disease but due to complexity and involvement of multiple players in the disease, persistence of inflammation even after the blockage of these single molecules has been observed (8).

Compound 7,4-dihydroxyflavone (DHF)- a flavonoid purified from *Glycyrrhiza uralensis* is one the most commonly used herbs in traditional Chinese medicine (TCM). We have shown that *G. uralensis* significantly inhibits TNF-α production (9). Likewise, we have highlighted key effects of DHF on suppression of Th2 cytokines-IL-4, IL-5 and IL-13 and serum IgE (10) and chemo attractant protein eotaxin/CCL-11 (11). The regulatory effect of DHF was further demonstrated in another study where DHF inhibited MUC5AC mRNA and protein expression (12) Recently, we also showed a case study of an 11 yr. old boy with complete clinical and tissue remission of EoE with TCM therapy, including Digestion teas (13). Based on our previous findings, we believe that DHF improvement of symptoms of EoE may be due to its anti-inflammatory effect, which ultimately reduces GI smooth muscle spasm, reverses the tissue inflammation, facilitates epithelium repair, and restores GI bacterial homeostasis.

**Figure 1:**
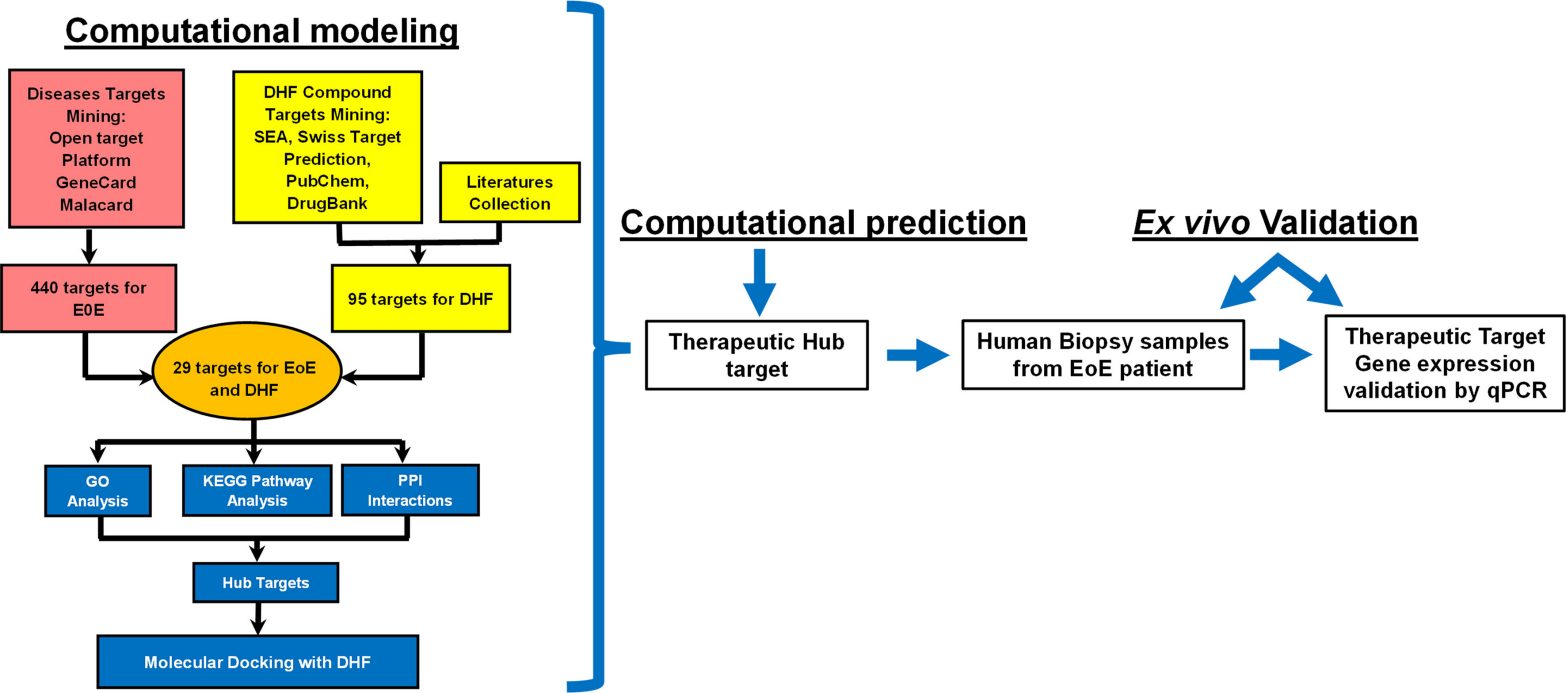
The workflow of computational modeling used for analysis of DHF as one of the promising candidates for Eosinophilic Esophagitis. First, total 440 target genes for EoE were selected. Likewise, 95 biological targets of DHF were collected based on literatures and following published databases including Swiss target prediction, SEA, pub-chem and drug-bank. Mining DHF targets into obtained disease targets uncovered that DHF might potentially regulate 29 targets for EoE. The GO, KEGG pathway and PPI analysis were further conducted to uncover the regulated details of DHF. Moreover, the hub proteins were determined for further molecular docking analysis. Furthermore, the therapeutic hub targets predicted by the computational modeling were validated *ex-vivo* in human esophageal biopsy samples from patients with EoE by ELISA and qRT-PCR. DHF: 7,4 dihydroxy flavone; SEA, similarity ensemble approach; PPI, protein-protein interactions, GO, gene ontology; KEGG, Kyoto encyclopedia of genes and genomes; EoE, eosinophilic esophagitis.

Given the complexity of pathological mechanisms of EoE and multiple targets of DHF on immunological responses, we utilized computational modeling—target mining, gene ontology enrichment, protein-protein interaction analyses, and *in silico* molecular docking to estimate the potential therapeutic mechanisms underlying the efficacy of DHF in EoE. The entire workflow of the study is shown in **Figure 1**. Guided by computational defined therapeutic targets, we employed human biopsy samples obtained from esophagus of EoE patients and determined DHF effects on those targets by tissue culture, ELISA, and qRT-PCR. The approaches in combination of computational modeling and *ex vivo* biological validation allowed us for the first time to identify potential therapeutic targets of DHF for EoE. This study provides guidance for our future *in vivo* experimental studies and future clinical trials.

## Materials and methods

### Target mining

Biological targets of DHF were identified from literature reports (14, 15) and published databases including HitPick (16), Swiss Target Prediction (17, 18), Similarity Ensemble Approach (19, 20), PubChem (21, 22), PharmMapper (23), and DrugBank (24, 25). The relevant human genes associated with EoE were selected as drug targets from various databases including Therapeutic Target Database (26, 27), Malacards (28), GeneCards (29, 30), and Open Targets Platform (31, 32). To ensure the predominance of targets, only the top 300 genes in each database were considered. Selected targets were finally mapped to UniProt Database (33, 34) for normalization. Next, the shared targets of DHF with EoE were obtained and these were considered to be potentially regulated targets of DHF for the management of patients with EoE.

### Gene ontology (GO), pathway and protein-protein interaction (PPI) analysis

Target enrichment gene ontology, pathway, and protein-protein interaction (PPI) analyses provided a molecular-level mechanistic insight into biological function. GO was introduced by mapping potential targets to the DAVID database (35, 36). The GO biological process terms with a false discovery rate of (FDR) <0.01 were selected. Pathways were obtained by mapping targets to KOBAS 3.0 (37, 38) and the significant pathways with FDR <0.01 were selected. Potential targets were mapped to String database, obtaining their interaction. The protein interactions were further used to construct the PPI network using Cytoscape (v3.2.1).

### Compound-target-pathway-disease network construction and analysis

With obtained targets and significant pathways, C-T-P-D biological networks were constructed using Cytoscape (v3.2.1). This network, containing DHF, its related targets for EoE, and significant principal pathways were constructed to comprehensively elucidate the complex relationship among compound, targets, and disease related pathways. This analysis provided general information about pharmacological mechanisms of DHF for the treatment and management of EoE at a molecular level. The properties of C-T-P-D networks were validated by NetworkAnalyzer (39), a plugin of Cytoscape.

### Molecular docking analysis

The binding modes of DHF with critical targets were predicted through molecular docking by AutoDock Vina (40). Protein crystal structures including IL1β (PDB:5R85), IL6 (PDB: 1ALU) (41), TNF (PDB:2AZ5) (42), and CCND1 (PDB:2W96) with excellent resolution were obtained from RCSB protein data bank (43, 44). The structure of DHF was directly downloaded from PubChemwithout further optimization. The molecular graphics were displayed by PyMOL system (45) (46), and Discovery Studio (47). Generally, all hydrogens and Gasteriger charges were added to each molecule. Docking areas and Autogrid parameters were set based on the binding pockets of proteins.

### Molecular dynamic simulation

The molecular dynamic simulations were carried out by Groningen Machine for Chemicals Simulations (GROMACS) with amber99sb-ildn force field and tip3p water model. 50 ns molecular dynamics simulation was performed for protein-ligand complex. The Root Mean Square Deviation (RMSD) analysis, and the toot-mean-square fluctuation (RMSF) were carried out using Xmgrace software.

### Esophageal biopsy culture

Pediatric subjects (aged 0-18 years old) with potential or known EoE were recruited under an IRB approved protocol at Mount Sinai Medical Center when undergoing an endoscopy as part of their routine clinical care. All subjects were determined to have active EoE based on clinical criteria including symptoms consistent with EoE and biopsies showing >15 eosinophils per high power field. Subjects ‘characteristics are listed in **Table 1**.

**Table 1:** Subject demographics.

Patient	Age (yr.)	Date of diagnosis	Sex	t-IgE (IU/L)	Food specific IgE	Treatment	Other allergies	Other conditions
P1	6Y	8/7/2018	F	–	Almond, milk, wheat, treenut, sesame, fish, shellfish, poppy	None	Allergic rhinitis	none
P2	8Y	8/2/2018	M	–	Egg white, egg yolk, pea, walnut, milk, almond, chickpea	budesonide	Asthma, allergic rhinitis, atopic dermatitis	None
P3	17Y	5/17/2018	F	–	Tree nuts (walnut, almond, brazil, cashew, pecan, macadamia), peanut	Omeprazole	Food allergies (peanut and tree nut), asthma	None
P4	5Y	9/8/2018	F	–	All negative	Omeprazole and milk avoidance	Asthma	None
P5	17Y	7/10/2018	M	–	All negative	omeprazole	None	GH Deficiency
P6	10Y	8/28/2018	F	–	All negative	None	Asthma, allergic rhinitis	None
P7	4Y	11/1/18	M	–	Tree nuts, salmon, beef	Protein Pump Inhibitor, TCM	Atopic dermatitis, allergic rhinitis	None
P8	10Y	8/24/2019	M	8	All negative	Fluticasone	None	celiac

TCM, Traditional Chinese Medicine; GH, Growth hormone; t-total.

Biopsies were digested with DNases I and Collagenase IV as previously described48 and divided equally into two wells then cultured with or without DHF (0.02mg/ml) in complete RPMI with 10% FBS overnight. Cytokines were measured by ELISA (BD Biosciences, NJ) as per the manufacturer’s instructions.

### Quantitative real-time PCR

RNA was extracted from precipitation of esophageal biopsy culture using TRIZOL (Invitrogen, Carlsbad, CA) followed by isopropanol precipitation. RNA was then reverse transcribed to complementary DNA (cDNA) using a PrimeScriptTM RT Reagent Kit (TaKaRa, Mountain View, CA). Real-time PCR was performed using SYBRTM Green Master Mix (Thermo Fisher Scientific, Fair lawn, NJ) as previously described (48). The target gene mRNA expression was normalized to the control group and calculated using the ΔΔCT method. The primer sequences are shown in **Table S1**.

## Results

### Target mining identifies the shared biological targets between DHF and EoE

**Figure 2:**
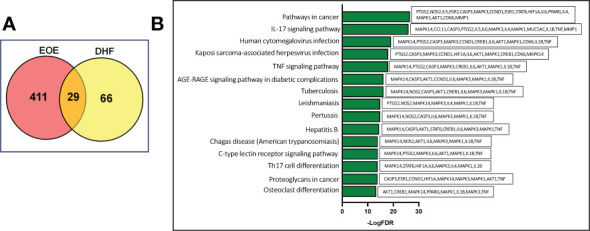
Computational identification and KEGG pathway analysis of the therapeutic targets. **(A)** Venn diagram showing the number of targets predicted using computational modeling. Among them 29 targets were shared between DHF and EoE. **(B)** KEGG Pathway analysis. Y-axis: The top 15 pathway relevant to the enriched targets (left) and genes associated with each pathway (right); X-axis: the significance of each term ranked with –log FDR. DHF, 7,4-dihydroxyflavone, EoE, Eosinophilic Esophagitis, FDR, False discovery rate.

The Venn diagram shows that 440 target genes for EoE were selected. Likewise, 95 biological targets of DHF were collected from the literature (14, 15), and published databases. Among them, 29 shared targets between DHF and EoE were discovered, which were finally selected as the potential therapeutic targets of DHF in the treatment and management of EoE (**Figure 2A**).

### Pathway analysis reveals complex signal transduction regulated by DHF

Further, to clarify the potential pharmacological effects of DHF on EoE from the pathway level, the pathway enriched analysis was performed by connecting the predicted targets to DAVID database. The top 15 significant KEGG pathways with False Discovery Rate (FDR) <0.01 and genes associated with each pathway were ranked and picked out by enrichment score –log (FDR) as shown in **Figure 2B**. Most pathways were related to host immune responses to inflammation and included “IL-17 signaling pathway”, “TNF-signaling pathway”, “AGE-RAGE signaling pathway in diabetic complications” “TH17 cell differentiation”, “Tuberculosis”, “Leishmaniasis”, “C-type lectin receptor signaling pathway” and “Pathway in cancer”. One of the main features of EoE is the accumulation of activated eosinophil at the site of inflammation. Eosinophils, generally, have a short life span of about 4 days and prolonged survival of eosinophil at the site of inflammation is mediated by IL-5, a potent type 2 cytokine (16). IL-5 along with IL-3 and GM-CSF mediate prolonged eosinophilic survival and release their cationic granular proteins, oxygen radicals and lipid mediators to cause tissue damage (16). Similarly, other pathways like “Human cytomegalovirus infection”, “Hepatitis B”, “Kaposi sarcoma-associated herpesvirus infection”, “Proteoglycans in cancer”, and “osteoclast differentiation” are involved in cell survival, proliferation, and progression. Overall, multiple pathways associated with EoE that were regulated by DHF were identified.

### Gene ontology reveals potential regulation of DHF in inflammation, apoptosis, oxidation, and transcription factor activity

The GO biological process terms in DAVID database were obtained with the identified targets as an enriched gene-set. The top 15 biological processes GO terms with False Discovery Rate (FDR) <0.01 were ranked by enrichment score (–log FDR) (**Figure S1**). The most significant GO biological process terms were closely associated with anti-apoptotic, anti-inflammatory, and anti-oxidative and anti-tumor properties. The most important pathways associated with these properties were “positive regulation of specific DNA binding transcription factor activity”, “positive regulation of transcription DNA-template”, “lipopolysaccharide-mediated signaling pathway”, “positive regulation of nitric oxide biosynthesis”, “positive regulation of transcription from RNA polymerase II promoter”, “inflammatory response”, “positive regulation of fever generation”, and “activation of MAPK”. Interestingly, most of the genes associated with these pathways (**Figure S1**) were inflammatory genes and were consistent with the KEGG pathway analysis. This further allowed us to explore the mechanism of DHF in treating EoE with respect to associated processes and function.

### Compound-target-pathway-disease network construction to select the crucial proteins

**Figure 3:**
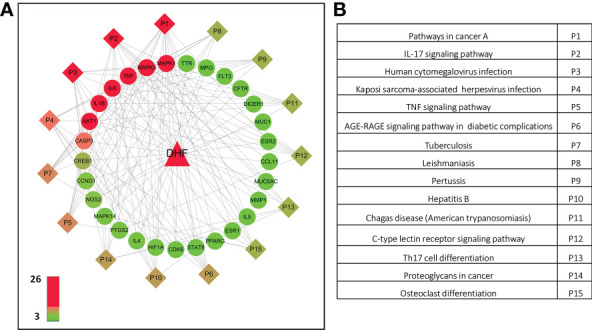
Compound-target- pathway-disease network of DHF for EoE. **(A)** By computational modeling, the role of DHF was deciphered through the drug-target network, and the target-disease network from the molecular level to the systems level. Triangle, circle, and diamond represent DHF, key targets and pathways, respectively. Node color from green to red is proportional to its degree. Black lines stand for interaction between the nodes The most important targets were TNF, MAPK1, MAPK3, IL6, IL1B, AKT1 and CASP3 (Red). The most important pathways associated with the protein targets were “Pathways in cancer” (P1), “IL-17 signaling pathway” (P2), and “Human cytomegalovirus infection” (P3). **(B)** Pathways associated in the compound-target-disease network.

The C-T-P-D network containing DHF, selected targets, top 15 pathways and EoE was developed to interpret the potential pharmacological mechanisms of DHF for management and treatment of EoE at the molecular level (**Figures 3A, B**). The C-T-P-D network provides general information about the complex interactions of compound, targets, and their related diseases. The frequency of targets appearing in the top 15 pathways implies their influence and importance. Node color from green to red is proportional to degree value, displaying importance from high to low in the network. The most important protein targets based on its degree from the C-T-P-D network were TNF, MAPK1, MAPK3, IL6, IL1B, AKT1 and CASP3 and the most important pathways associated with these protein targets were “Pathways in cancer”, “IL-17 signaling pathway”, and “Human cytomegalovirus infection”. Overall, the C-T-P-D network helped to simplify the complex interaction between proteins, and pathways associated with EoE and DHF. Furthermore, it helped to select key target proteins which could potentially be targeted by DHF in treating EoE.

### Protein-protein interaction network construction to confirm the vital function of proteins

**Figure 4:**
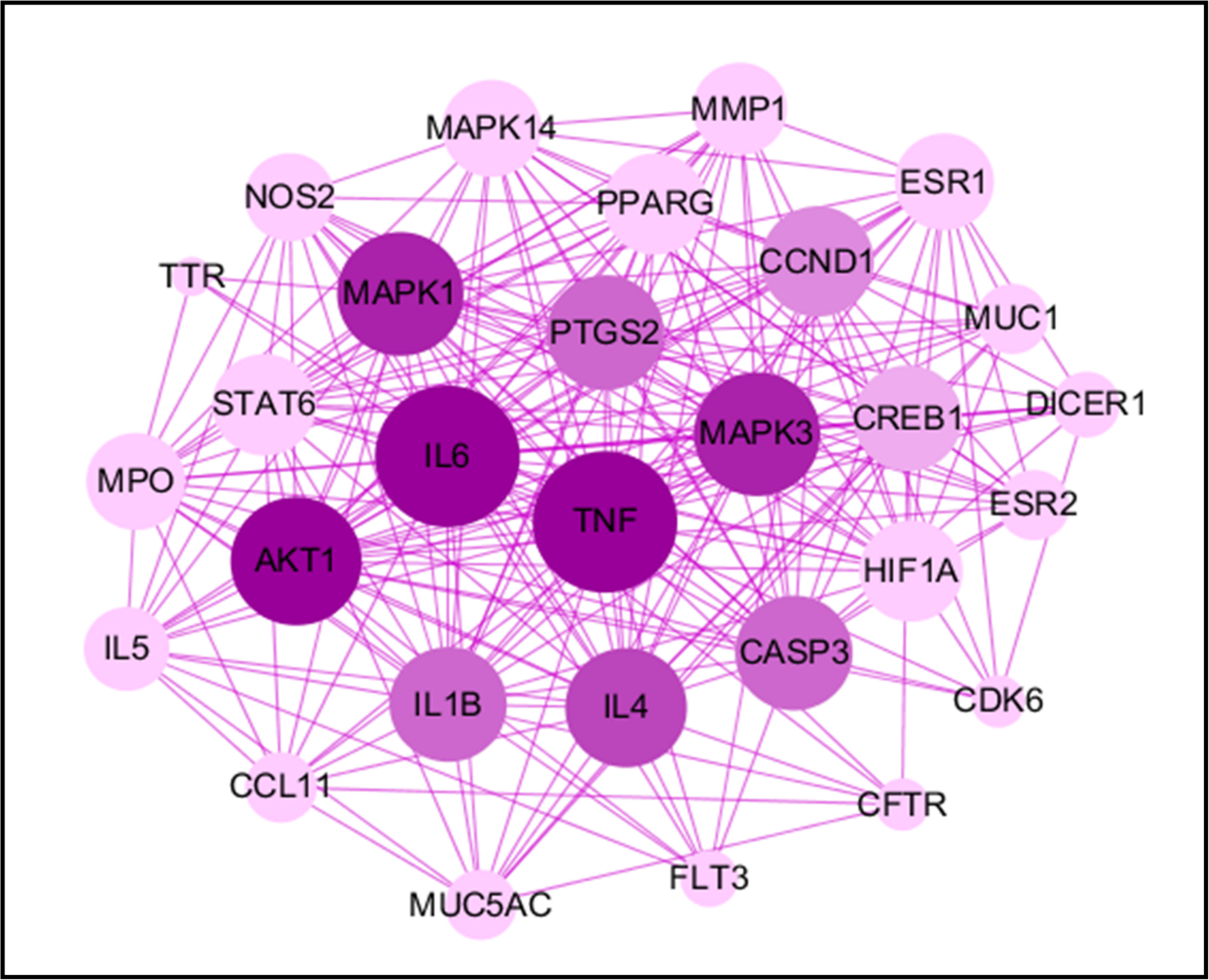
Protein-protein interactions. The PPI network was constructed by mapping potential targets to the Strings database. The size of the node from large to small is proportional to its degree value in the network. The circles represent the therapeutic targets, and the purple lines represent the interaction between the nodes.

The PPI network was constructed by mapping potential targets to the String database (17). The size of the node from large to small is proportional to its degree value in the network. It is well known that protein-protein interactions are critical to a wide range of biological processes, including cell-to-cell interaction and metabolic and developmental control (18). A deeper understanding of such complex relationships among disease-related proteins provides new opportunities to investigate the molecular mechanisms of diseases (19). Recently, PPI has become a reliable tool to evaluate protein functions in the network and determine hub proteins in the regulation of diseases. In this study, we found TNF occupying the central position in the network along with IL6, MAPK3, MAPK1 and AKT1 (**Figure 4**). The branching lines from these proteins represent its interaction with other proteins and we found highest interaction of these key proteins with others in the protein-protein interaction analysis. The other important proteins in the network were IL1B, IL4, CASP3, CCND1, and PTGS2 and these proteins showed moderate rank in the PPI network. Some of the less important proteins formed the outer layer of the network and showed lowest interaction with others. Overall, the PPI network led to specifically select key proteins with the highest interaction index to better understand how DHF could potentially regulate these proteins in management of EoE.

### Molecular docking analysis and dynamic simulation predicts the binding modes between DHF and its crucial targets

**Figure 5:**
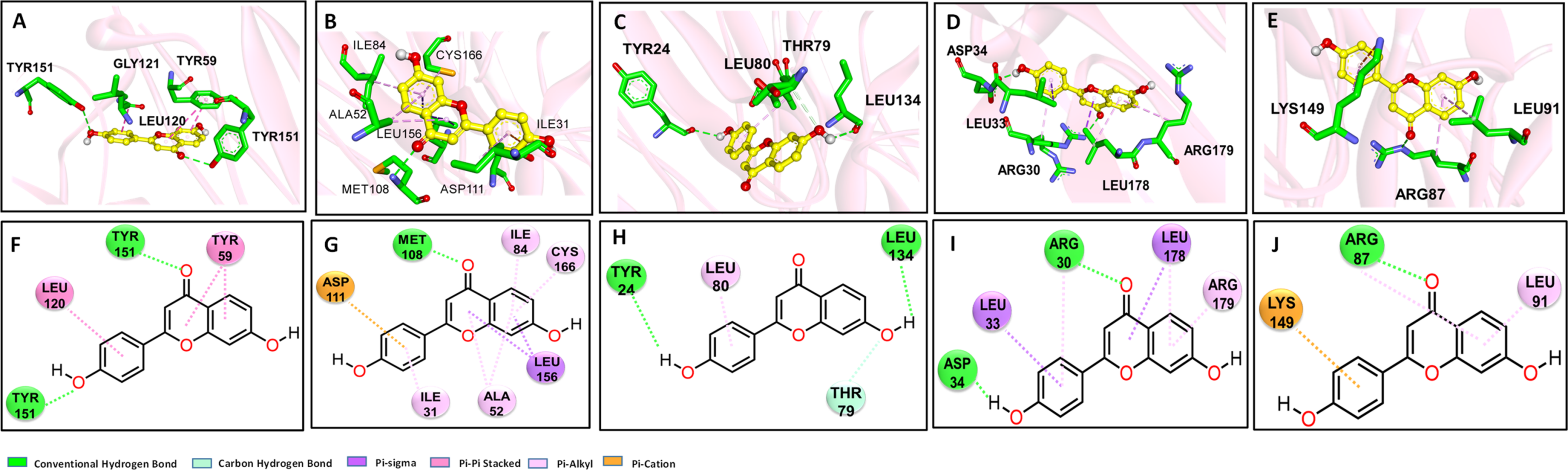
Molecular docking analysis to predict the binding modes between DHF and its therapeutic targets. Based on the gene expression results in the esophagus, the binding explorations of complex MAPK1-DHF, IL1B-DHF, IL6-DHF, TNF-DHF, CCND1-DHF were explored. Predicted lowest-energy binding mode of DHF in 3-dimentiaonal figure with the following proteins: **(A)**, **(F)** TNF-α; **(B)**, **(G)** MAPK1; **(C)**, **(H)** IL1B; **(D)**, **(I)** IL6; **(E)**, **(J)** CCND1; the carbon, oxygen and nitrogen are highlighted in yellow, red, and blue, respectively. For residents of proteins, the green, red, and blue stand for C, O and N, respectively.

We assumed that DHF regulated the crucial targets by interfering in gene expression or binding with proteins directly. Therefore, a molecular docking was introduced to calculate the binding energy and evaluate binding favorability between DHF, and crucial targets. The binding energy indicated that DHF might be a promising regulator for each of the selected targets with binding affinities between -6.3 to -7.7 Kcal/mol (**Table S2**). The optimal binding modes of DHF with targets including TNF-α, MAPK1, IL1B, IL-6, and CCND1 is shown in **Figure 5**. The best results were obtained for complex TNF-DHF with a free binding energy of -7.7 kcal/mol. The Hydrogen bonds between DHF with TYR151 and the π-π stacking between DHF and LEU120 stabilized the left structure of DHF in one chain (**Figures 5A, F**). Likewise, DHF displayed similar binding affinities with MAPK1 and IL1-β. For MAPK1-DHF complex, the hydrogen bond (MET108), π-alkyl interactions (ILE31, ILE84, ALA52, CYS166), and π-anion interactions (ASP111) with DHF stabilizing the configuration of DHF in the complex (**Figures 5B, G**). Similarly, for complex IL1B-DHF, the hydrogen bond between DHF and residues (TYR24, LEU134, THR79) significantly contributed to the stability of the complex (**Figures 5C, H**). For complex IL6-DHF, DHF fitted well in the binding cavity of IL-6 surrounded by hydrogen bonds (ASP34, ARG30), π-sigma (LEU33, LEU178), π-alkyl (ARG179) and π-π (ARG30) interactions (**Figures 5D, I**). Lastly, for the complex CCND1-DHF, the residues ARG87, LEU91, and LYS149 facilitated the binding interactions by hydrogen, π-π stacking, and π-cation effects (**Figures 5E, J**). To evaluate the stability of binding, TNF-DHF complex were further optimized by dynamic stimulation using Gromacs. The stability and flexibility of complex were estimated by RMSD (**Figure S2A**) and RMSF (**Figure S2B**) analysis respectively. The trend of RMSD figure indicated the stable binding of DHF with TNF. It is worth noting that the average RMSD value for TNF was 4.9 Å. The RMSF analysis implied minor deviation of protein during the simulation period. The most stable binding mode of TNF-DHF were displayed in **Figures S2C, D** respectively. Residues TYR59, ILE155, and LEU57 contributed to maintain the stability of DHF with TNF. Overall, the molecular docking results indicated that DHF bind with the crucial proteins directly to regulate the resulting biological effects.

### DHF inhibited pro-inflammatory cytokine levels and its expression *in- vitro* in human esophageal biopsy culture

**Figure 6:**
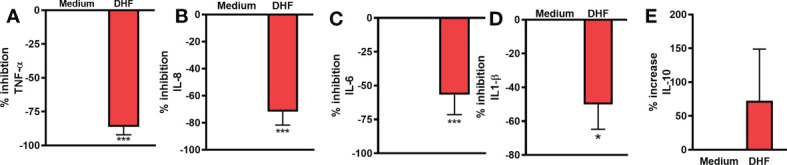
Anti-inflammatory effect of DHF on esophageal biopsy sample: Esophageal biopsy samples were cultured with or without DHF for 24 hrs. and cytokine levels in the culture supernatant were measured by ELISA. Percentage inhibition of cytokines TNF-α **(A)**, IL-8 **(B)**, IL-6 **(C)**, IL1-β **(D)** and percentage of increase in cytokine IL-10 **(E)** in presence of DHF were analyzed. Data represents Mean ± SEM. N=6-8 samples. ***p < 0.001; *p < 0.05 compared to Medium. Paired *t* test was used for statistical analysis.

**Figure 7:**
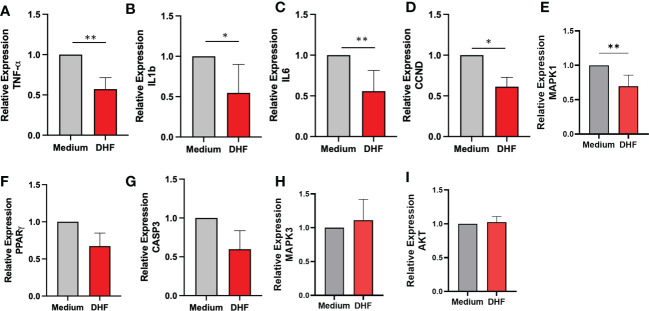
Effect of DHF on therapeutic target gene in esophageal biopsy sample: Based on the therapeutic hub targets predicted by the computational modeling, the expression levels of key targets were validated *ex-vivo* using human esophageal biopsy samples cultured in the presence of DHF. The relative expression levels of TNF-α **(A)**, IL1β **(B)**, IL-6 **(C)**, CCND **(D)** and MAPK1 **(E)** were significantly inhibited in the presence of compound DHF. Similarly, the expression levels of PPARγ **(F)**, CASP3 **(G)**, MAPK3 **(H)** and AKT **(I)** showed no significant difference as compared to the medium. Data represents Mean ± SEM. N = 6-8 samples. *p < 0.05; **p < 0.01 compared to Medium. Paired *t* test was used for statistical analysis.

To provide experimental support and validate the claim that DHF could be an effective alternative for the management of EoE patients, we evaluated the effect of DHF on key therapeutic targets obtained from the computational analysis. We evaluated the levels of different pro-inflammatory cytokines by ELISA and their expression by qRT-PCR in human esophageal biopsy cultures with or without the presence of DHF. In presence of DHF, we found significantly lower levels of pro-inflammatory cytokines- TNF-α, IL-8, IL-6, IL-1B respectively (**Figures 6A–D**) and slight increase in IL-10 levels (p=0.38) (**Figure 6E**) in the culture supernatant of esophagus biopsies. Furthermore, in presence of DHF, the gene expression analysis showed marked decrease in the expression levels of TNF-α, IL1-B, IL-6, CCND and MAPK1 respectively (**Figures 7A–E**). Likewise, there was a slight decrease in expression of CASP3 and PPARγ (p=0.08) respectively (**Figures 7F, G**). However, there was no statistical differences in the expression levels of MAPK3 and Akt respectively (**Figures 7H, I**).

## Discussion

In this study we, for the first time, used a computational approach that integrated target mining, network technology and pathway analysis to reveal the potential therapeutic pathways and targets to understand pharmacological mechanisms of DHF in treating EoE. The cutting-edge technologies allowed us to uncover key mechanisms involved in the pathogenesis of EoE and further advance our knowledge into new therapeutic applications. It is believed that mechanism of EoE is strictly driven by complex interplay between genetic and early life environmental risk factors (20), interaction and signaling between epithelial, mesenchymal, and immune cells on molecular and intracellular level (21). Based on our results, we selected top 15 pathways of which majority of them were inflammatory. This further adds to the complexity of the disease by deciphering key inflammatory molecular pathways and provide evidence that these pathways may be contributing directly or indirectly to the progression of disease. Some of the important pathways were- pathway in cancer, IL-17 signaling, TNF signaling, AGE-RAGE signaling in diabetic complications, and several other infectious disease related pathways. Furthermore, by mapping these pathways in the C-P-T-D network, we identified the key targets associated with these pathways which could potential be regulated by DHF in EoE. The most prominent targets were MAPK1, MAPK3, TNF, IL6, IL1B and AKT1, also known to as an important cytokine regulating tissue damage. The pathogenesis of EoE is still incompletely understood but it be believed to be a consequence of a complex interplay between genetic, environment and host immune cells (22). Beside these factors, it is plausible that the pathways and genes identified in this study by computational approach could be playing a role in the pathogenesis of EoE. Further studies are necessary to better understand the mechanisms at molecular level. Similarly, the molecular docking and dynamic simulation analysis revealed that DHF can directly bind to the target molecules with similar binding affinities to regulate its action in preventing inflammation. The strongest binding with superior stability and minor deviation was seen between DHF and TNF. The Molecular simulation could accurately predict many important dynamics, but sometimes these simulations are not suitable to systems where metal atoms are involved in binding requiring robust quantum mechanical calculations. Finally, we validated our computational modeling results using esophagus biopsy sample from patient with EoE. We demonstrated that in presence of DHF there was significant reduction in the levels of TNF-α, IL-8, IL-6, IL1B and in the culture supernatant and further validated its effect in the molecular level by qRT-PCR. Our gene expression data showed significant decrease in the expression of key pro-inflammatory genes TNF-α, IL-1B, IL-6, CCND and MAPK1. This study provides an evidence DHF could potentially be regulating inflammation in EoE and further studies with large sample size should be conducted to better understand the mechanisms associated with it.

At present, dupilumab is the only the FDA approved treatment for EoE (23), and highlighting the complex nature of the disease, it is crucial to advance our knowledge and strengthen the research into therapeutic agents that can target multiple aspects of the disease. Evidence of successful management of a patient with GI symptoms secondary to EoE using digestion tea containing DHF (13) opens up the area to study the role of flavonoid in treating EoE. With this study, we were able to successfully identify key targets and subsequently validate it using the human esophagus biopsy sample from patient with EoE. It could be possible that DHF mechanism of acting in successfully treating EoE patient was due to its action on the pathways and genes that we identified in our study. This study may provide a potential role of DHF as an active therapeutic candidate in the management and treatment of EoE. The direct effect of DHF in animal models of eosinophilic disease is the next step towards understanding and determining if DHF will be efficacious for the treatment of EoE.

The current study uses computational approach to identify key targets which are potentially regulated by DHF in EoE. However, the key targets obtained using this approach are not primary drivers of eosinophilic esophagitis but based on the evidence on the literature the role of TNF in EoE has been actively investigated. It has been reported that the inflamed epithelial cells prime esophageal fibroblasts to secrete the profibrogenic cytokines IL-1β and TNF-α, which in turn promote epithelial-to-mesenchymal transition and esophageal fibrosis (48). Additionally, it has been reported that cross talk between esophageal epithelial cells and fibroblast leads to robust production of TNF, contributing to fibrostenotic EoE (49). Furthermore, several TNF superfamily of proteins has been shown to be transcribed by several different subsets of T cells in the esophagus including the pathogenic effector Th2 cells (50). This makes us further believe that TNF play an important role in EoE, possibly at the chronic stage and a therapy targeting TNF in EoE would potentially contribute to one endotype of EoE. With current therapy having limited effects on treating fibrostenotic EoE, and ample evidence of production of TNF by esophageal fibroblast to contribute to inflammatory cascade in EoE (51), this study opens up a new area in EoE therapeutics by showing the role of DHF in modulating pro-inflammatory markers in EoE. In addition, most of the targets that we found in this study shared common pathways of inflammation and it plausible that all these inflammatory players could possibly be playing a role in exacerbating eosinophilic inflammation. Even though the effect of DHF on predicted target using computational approach seems promising, further studies are required to test the effect of DHF clinically for proposed activity against inflammatory EoE as well as the detailed mechanism of action on how these genes and targets interact with primary drivers of EoE, including Th2 genes- IL-4, IL-5, and IL-13 needs to be studied. This may be done either alone or in combination with an FDA approved treatment, Dupixent.

## Data availability statement

The original contributions presented in the study are included in the article/**Supplementary Material**. Further inquiries can be directed to the corresponding author.

## Ethics statement

The studies involving human participants were reviewed and approved by IRB approved protocol at Mount Sinai Medical Center. Written informed consent to participate in this study was provided by the participants’ legal guardian/next of kin.

## Author contributions

All authors listed have made a substantial, direct, and intellectual contribution to the work and approved it for publication.
