# Supplementary material for: Computational analysis to define efficacy & molecular mechanisms of 7, 4’- Dihydroxyflavone on eosinophilic esophagitis: Ex-vivo validation in human esophagus biopsies
Source: Front Immunol. 2022 Dec 15;13:1015437. doi: 10.3389/fimmu.2022.1015437 (PMC9797535; doi:10.3389/fimmu.2022.1015437)
Supplement: Supplementary file 1 [file Table_1.docx]

| **h-Primer** | **Sequence (5’-3’)** |
| --- | --- |
| **TNF-α** | **F- CTCTTCTGCCTGCTGCACTTTG**  **R- ATGGGCTACAGGCTTGTCACTC** |
| **AKT1** | **F- TGGACTACCTGCACTCGGAGAA**  **R- GTGCCGCAAAAGGTCTTCATGG** |
| **IL1b** | **F- GCAGAGACTGTAGGTAGTTTCGG**  **R- GCAGAGACTGTAGGTAGTTTCGG** |
| **MAPK1** | **F- CCATCTGCCTTGCTTACCTT**  **R- AGCACCTGGGACTCAAACTG** |
| **IL-6** | **F- CCCTGACCCAACCACAAATGC**  **R- CAACAACAATCTGAGGTGCCCAT** |
| **MAPK3** | **F- TGGCAAGCACTACCTGGATCAG**  **R- GCAGAGACTGTAGGTAGTTTCGG** |
| **CASP3** | **F- TGATGATGTGGAAGAACTTAGG**  **R- ACGGCTCCGCACCTGCTGAGGC** |
| **CCND** | **F- ATGTTCGTGGCCTCTAAGATGA**  **R- CAGGTTCCACTTGAGCTTGTTC** |

Table S1: Primer sequence used in the study

Table S2: Molecular docking result between relevant proteins and DHF

| **Compound** | **Gene Name** | **Affinity (Kcal/mol)** |
| --- | --- | --- |
| DHF | TNF | -7.7 |
|  | MAPK1 | -7.3 |
|  | IL1B | -7.3 |
|  | IL6 | -6.9 |
|  | CCND1 | -6.3 |

Table S2: The binding modes of DHF with critical targets were predicted molecular docking by AutoDock Vina. The higher negative binding score indicate that DHF binds to the selected target with a higher affinity as indicated.
