# Supplementary material for: Computational analysis to define efficacy & molecular mechanisms of 7, 4’- Dihydroxyflavone on eosinophilic esophagitis: Ex-vivo validation in human esophagus biopsies
Source: Front Immunol. 2022 Dec 15;13:1015437. doi: 10.3389/fimmu.2022.1015437 (PMC9797535; doi:10.3389/fimmu.2022.1015437)
Supplement: Supplementary file 2 [file Presentation_1.pptx]

## Slide 1
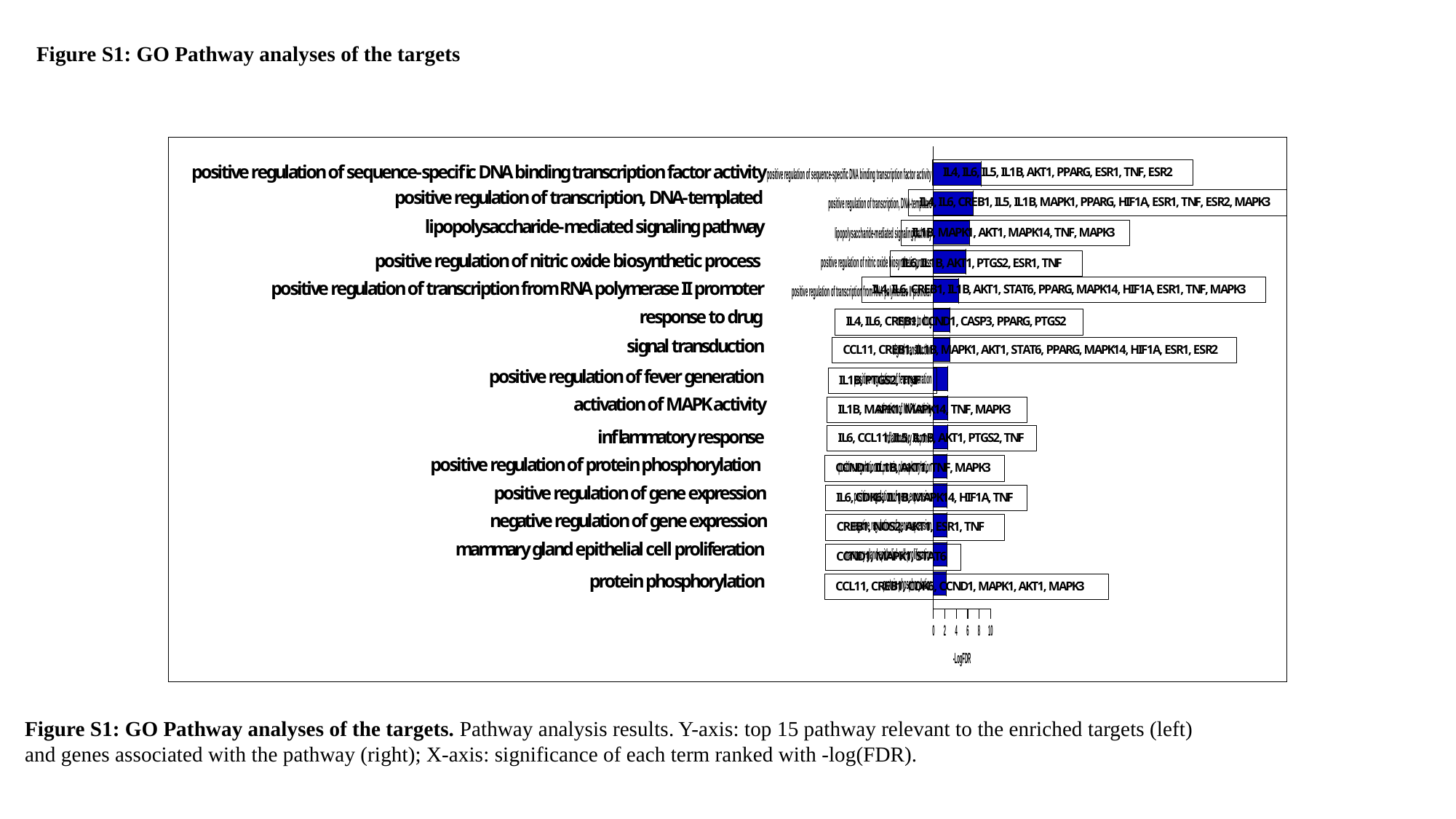

Figure S1: GO Pathway analyses of the targets
Figure S1: GO Pathway analyses of the targets. Pathway analysis results. Y-axis: top 15 pathway relevant to the enriched targets (left) and genes associated with the pathway (right); X-axis: significance of each term ranked with -log(FDR).

## Slide 2
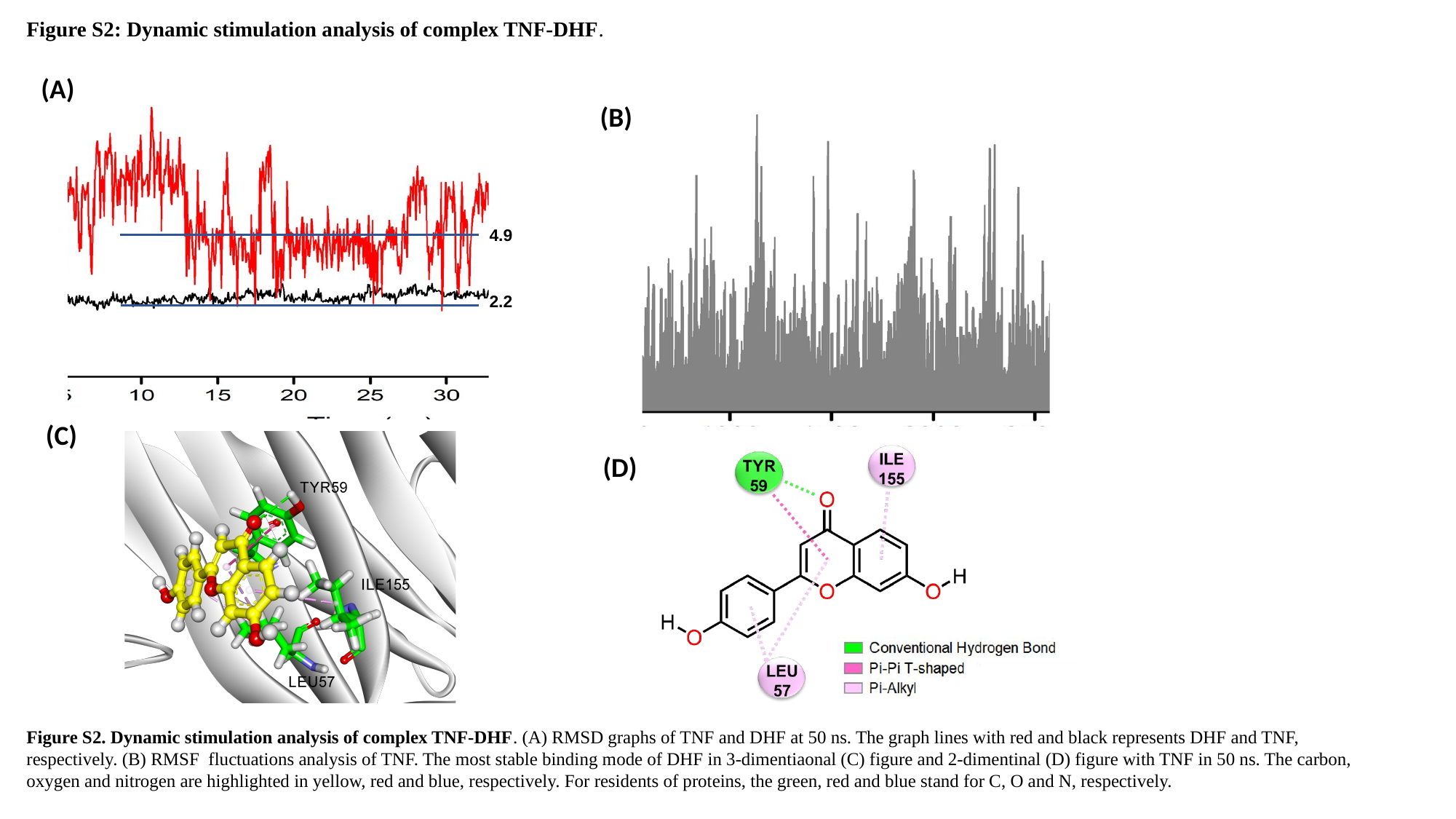

Figure S2: Dynamic stimulation analysis of complex TNF-DHF.
(A)
(B)
4.9
2.2
(C)
(D)
Figure S2. Dynamic stimulation analysis of complex TNF-DHF. (A) RMSD graphs of TNF and DHF at 50 ns. The graph lines with red and black represents DHF and TNF, respectively. (B) RMSF fluctuations analysis of TNF. The most stable binding mode of DHF in 3-dimentiaonal (C) figure and 2-dimentinal (D) figure with TNF in 50 ns. The carbon, oxygen and nitrogen are highlighted in yellow, red and blue, respectively. For residents of proteins, the green, red and blue stand for C, O and N, respectively.
